# Jejunogastric Invagination Complicated With Intestinal Necrosis

**DOI:** 10.7759/cureus.97593

**Published:** 2025-11-23

**Authors:** Pedro Nel Montoya Restrepo, Daniela Gomez, Luisa Meza Botero, Ana Fernanda Muñoz Durán, Leidy Aguirre

**Affiliations:** 1 Radiology, Hospital Manuel Uribe Angel, Envigado, COL; 2 General Medicine, Universidad de Manizales, Manizales, COL; 3 General Medicine, Manizales University, Manizales, COL; 4 General Medicine, Fundación Universitaria San Martín, Sabaneta, COL; 5 General Practice, Universidad de Antioquia, Medellín, COL

**Keywords:** bowel ischemia, digestive surgery, gastric complications, jejunal intussusception, small bowel necrosis

## Abstract

Jejunogastric intussusception (JGI) is an uncommon complication of gastric surgery. Its causes are varied, and it is classified as acute or chronic depending on the time of onset. The clinical presentation is usually nonspecific, with symptoms such as epigastric pain, nausea, vomiting (with or without blood), and signs of upper gastrointestinal obstruction, which can be mistaken for neoplastic disease or peptic ulcer disease. The diagnosis is established by endoscopy or radiology, with computed tomography (CT) being particularly useful for defining the site of intussusception by demonstrating the typical findings of a target or pseudokidney appearance, as well as for quickly defining the extent and complications. Treatment usually requires urgent surgery to reduce the intestinal intussusception, assess the viability of the intestine, and, if necessary, perform an intestinal resection. Surgical outcomes depend on the duration of the condition, with a reported mortality rate of 5%-50% if treatment is delayed for more than 48 hours, a recurrence rate of up to 10% if the cause is not corrected, and prolonged hospital stays with extensive intestinal resections. We present a case of a patient with an unclear history of surgery for a perforated peptic ulcer complicated by JGI and intestinal ischemia.

## Introduction

Jejunogastric intussusception (JGI) is an infrequent but serious postoperative complication that occurs most frequently after gastrojejunostomy and other upper gastrointestinal reconstructions. Although rare, a growing number of case reports and reviews emphasize its diverse clinical presentations and diagnostic challenges. The clinical presentation ranges from severe acute abdominal pain and vomiting to upper gastrointestinal bleeding, including hematemesis [1-3]. Reported etiologies and settings for JGI include classic gastrojejunostomy-related cases as well as occurrences after more complex operations such as pancreaticoduodenectomy and distal gastrectomy performed for gastric cancer. Unusual patterns have been described, including retrograde (antiperistaltic) intussusception [4], double intussusceptions involving both jejunojejunal and jejunogastric components [5], and intussusception secondary to an intraluminal mass such as a gastrointestinal stromal tumor causing gastroduodenal involvement [6,7]. Because the invaginated jejunum may appear as an intragastric mass, JGI is sometimes misdiagnosed as gastric cancer on initial evaluation, delaying appropriate treatment. If not managed within the first 48 hours, mortality can be as high as 50% [3]. Advances in imaging have improved preoperative recognition in some reports, but since the evidence is based primarily on case reports, maintaining a high index of suspicion and prompt surgical evaluation remain crucial to reducing morbidity and mortality [3]. We present the unusual case of a patient with a JGI complicated by intestinal ischemia secondary to an unclear surgical history due to acid-peptic disease.

## Case presentation

A 71-year-old man was admitted with a three-day history of severe epigastric pain accompanied by nausea, episodes of hematemesis, and weight loss. He reported a remote laparotomy for a perforated peptic ulcer approximately 20 years earlier, although the surgical details were unclear. On admission, the patient appeared malnourished and pale. His vital signs showed hypotension (90/50 mmHg) and tachycardia (110 bpm), with no other vital signs altered. On examination, the abdomen was soft and nondistended, with tenderness on deep palpation in the epigastrium and mesogastrium, with no signs of peritoneal irritation. Laboratory tests showed an elevated C-reactive protein level of 7 mg/L, with no other significant findings.

**Figure 1:**
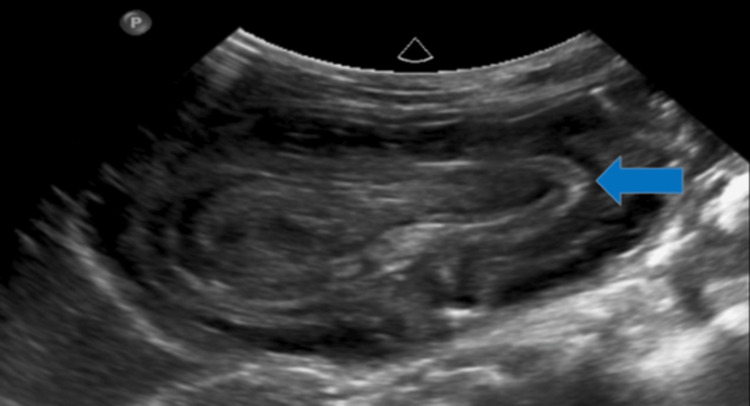
The blue arrow shows a kidney-shaped lesion inside the stomach, with a central hyperechoic portion or echogenic band surrounded by a hypoechoic region, which constitutes the pseudokidney sign.

**Figure 2:**
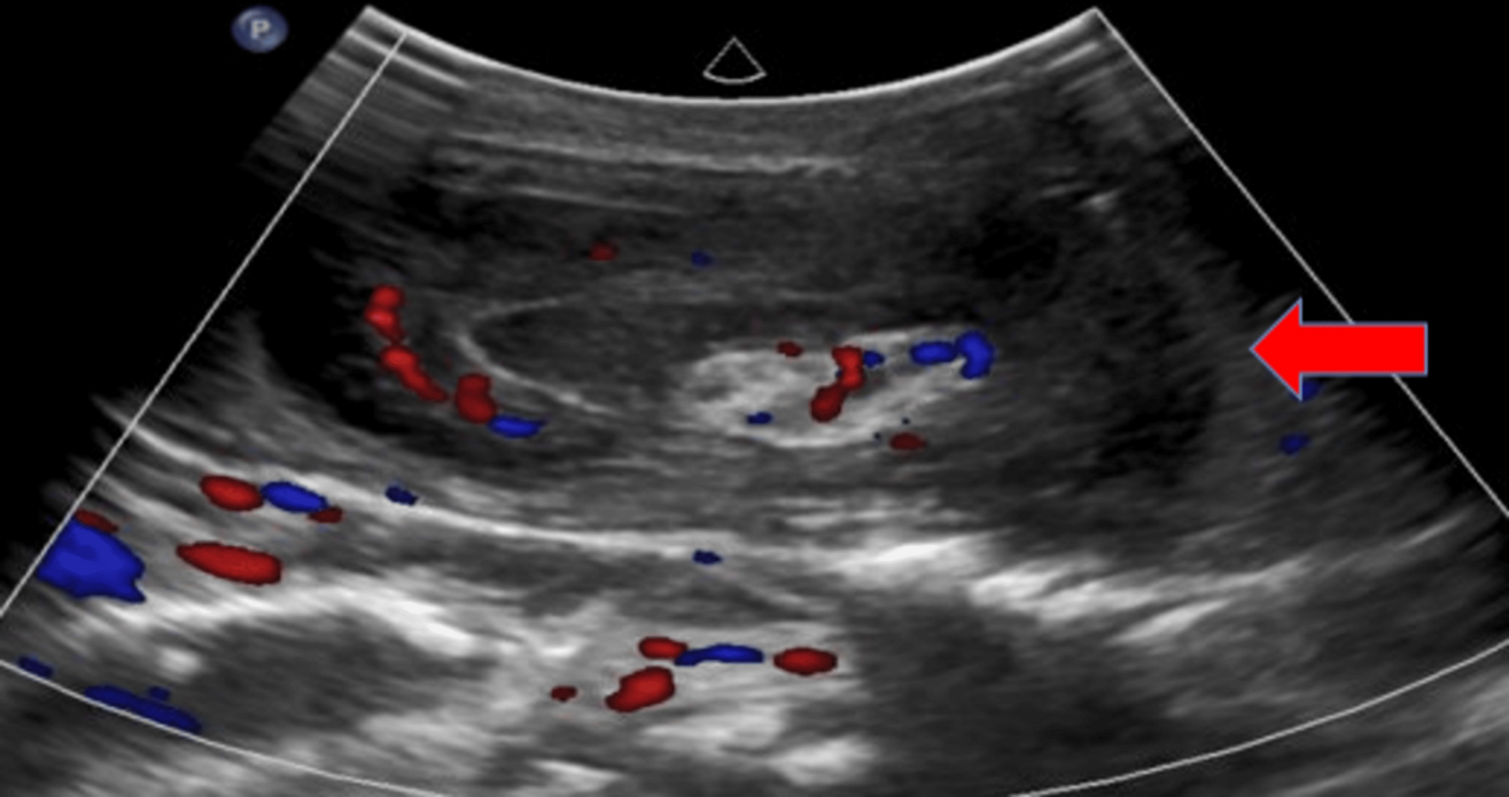
The red arrow shows concentric alternating echogenic and hypoechoic bands inside the stomach above the upper portion, forming the target sign.

An abdominal ultrasound performed at presentation showed a heterogeneous intragastric lesion with central echogenicity in the left hypochondrium, with alternating hypoechoic bands. The lesion had a target sign in its upper part and a false kidney appearance in its lower part; it showed peristaltic movement and poor vascularization on Doppler ultrasound (Figures 1, 2).

**Figure 3:**
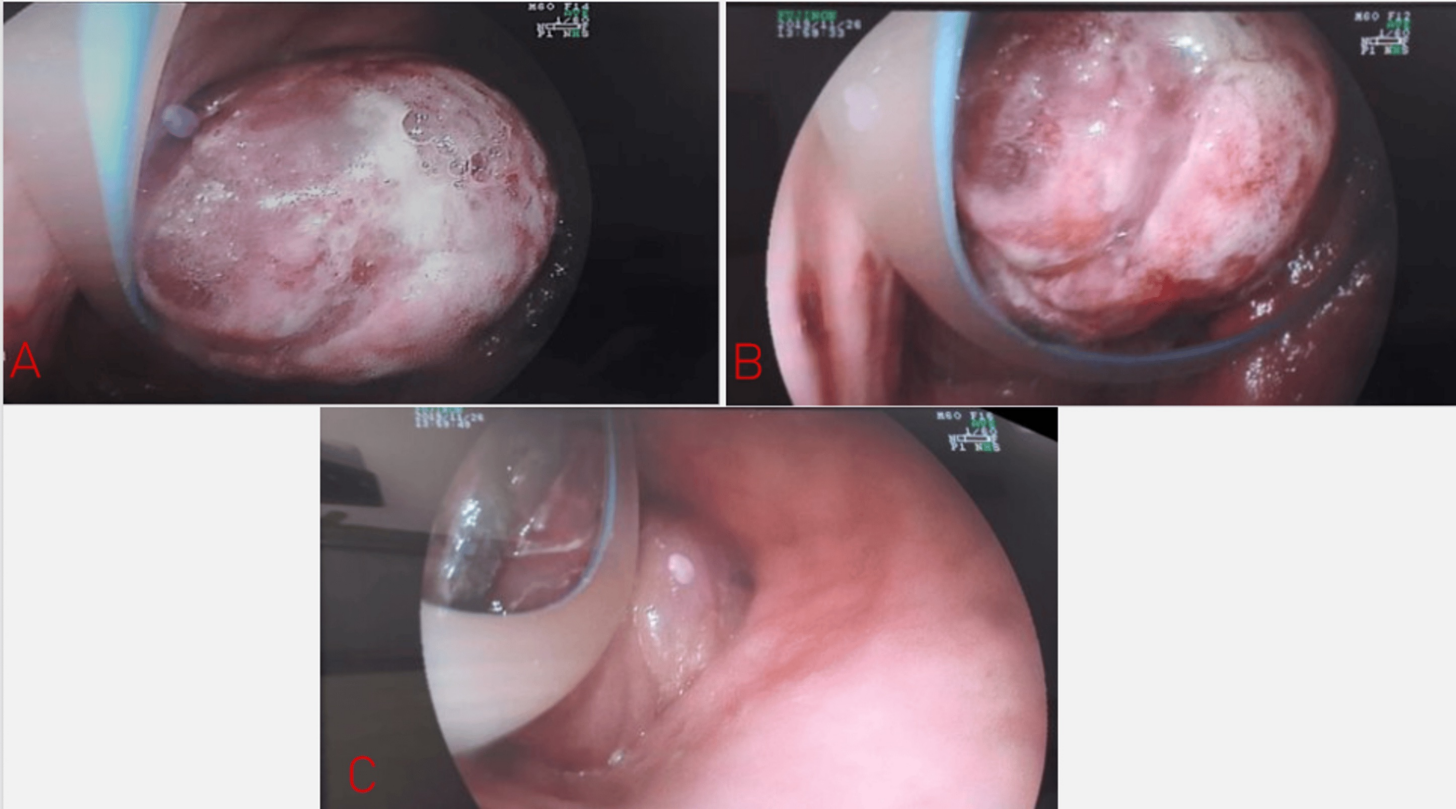
(A) Video endoscopy demonstrates intussusception of jejunal loops into the stomach. (B) Intussuscepted intestinal loops with an edematous and violaceous appearance. (C) Reduction of the intussusception was unsuccessful.

With an initial suspicion of intussusception secondary to advanced gastric cancer, an upper gastrointestinal endoscopy was performed one day after admission. The stomach was approximately 30% occupied by a purplish intragastric prolapse of the small intestine; the lumen of the prolapsed segment could not be identified (Figure 3). The endoscope was advanced through the pylorus to the duodenum, supporting the conclusion that the intussusception originated from a gastrojejunal anastomosis.

**Figure 4:**
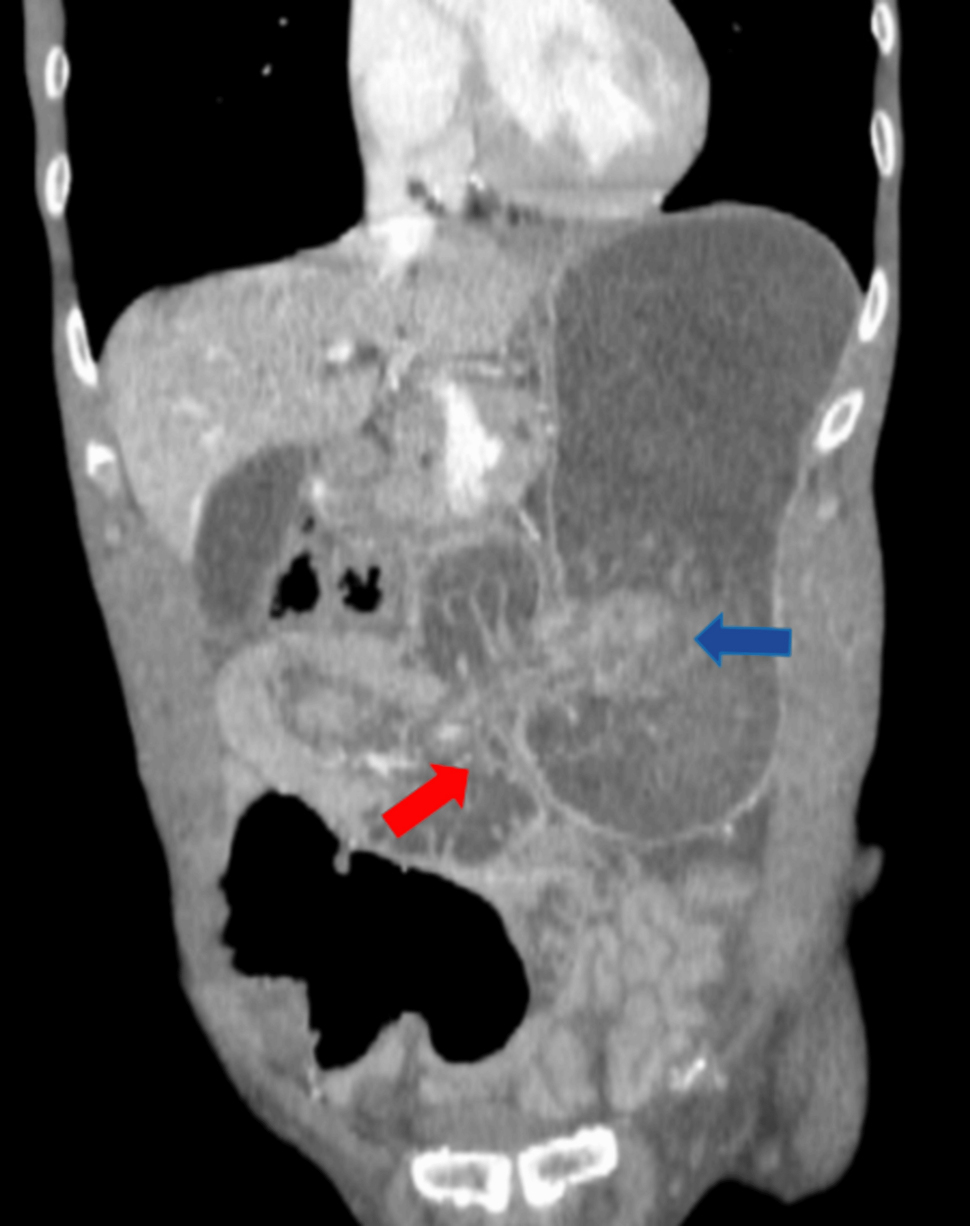
The coronal CECT image shows invaginated jejunal loops within the lumen of the stomach (blue arrow) via a defect in the anteroinferior wall of the stomach in the anteropyloric region (red arrow). CECT: Contrast-enhanced computed tomography.

**Figure 5:**
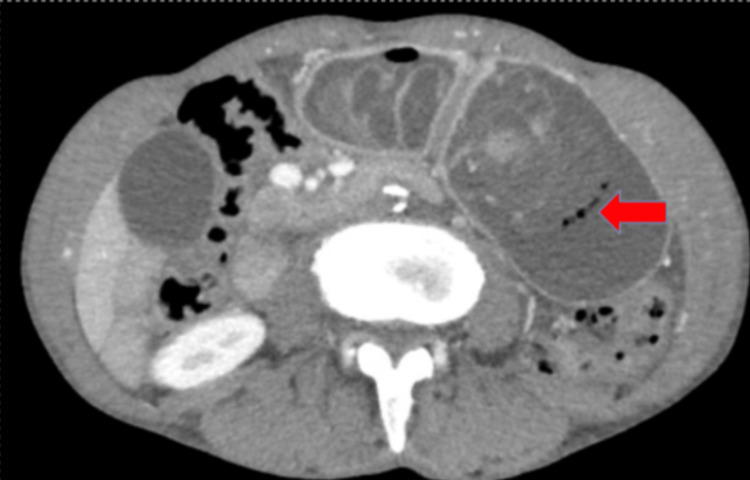
Axial CECT image shows invaginated jejunal loops into the stomach with pneumatosis within the wall suggesting bowel ischemia (red arrow). CECT: Contrast-enhanced computed tomography.

A contrast-enhanced abdominal CT scan was requested, which revealed a 38-mm mural defect in the greater curvature of stomach in the anteropyloric region in the axial and coronal slices with maximum intensity projection (MIP) reconstruction, through which loops of the jejunum were invaginated (Figure 4), some of which showed edema and absence of intestinal wall enhancement associated with signs of pneumatosis due to ischemia (Figure 5).

**Figure 6:**
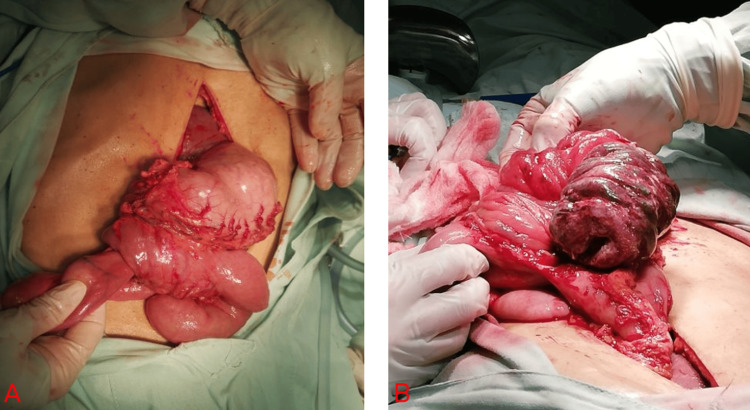
(A) Well-perfused stomach with a defect through which intestinal loops were invaginated. (B) Invaginated intestinal loops with signs of ischemia.

The patient underwent emergency surgery two days after arriving at the emergency room. An urgent midline laparotomy revealed a markedly dilated but well-perfused stomach and intussusception of the gastrojejunostomy, with a segment of jejunum approximately 20 cm prolapsed into the gastric lumen showing transmural ischemia (Figure 6). The intussusception was manually reduced, and approximately 30 cm of ischemic jejunum was resected. A term-to-term (end-to-end) jejunojejunal anastomosis was performed, and the gastric defect was repaired (gastrorrhaphy). After the operation, the patient had a slow recovery and required nutritional support for 19 days until discharge.

## Discussion

JGI is an atypical complication of gastrointestinal surgery, first described by Bozzi in 1914 in a patient who underwent gastrojejunostomy.

The etiology of JGI is not well understood. Proposed functional factors include hyperacidity, abnormal intestinal motility, and episodes of increased intra-abdominal pressure; the condition can also occur spontaneously [7]. Mechanical factors are often related to a history of gastrointestinal surgery, such as gastrojejunostomy, Billroth II gastrectomy, or Roux-en-Y reconstruction. The incidence after these operations has been reported to be less than 0.1% [3].

JGI is classified as acute or chronic. The acute form presents with severe abdominal pain and hematemesis. In the chronic form, recurrent episodes of gastric fullness and vomiting are described, resulting from intermittent and reversible intestinal intussusception. A classification of JGI has been described according to the invaginated loop. Type II with retrograde or efferent intussusception of the loop accounts for 80% of cases [8-10].

The diagnosis is established through imaging. Plain abdominal X-rays may show a markedly distended stomach with an intragastric mass. Contrast studies using a water-soluble agent may show a spring sign corresponding to jejunal invagination into the stomach. Abdominal ultrasound usually reveals a tubular or target-shaped intragastric mass with peristaltic movement, whereas CT provides detailed anatomical delineation and can detect intestinal ischemia. Although endoscopic reduction has been successfully performed in selected stable cases, higher recurrence rates have been reported [11,12], and surgical intervention remains the definitive treatment, especially in patients with signs of ischemia, shock, or peritonitis. If not treated within the first 48 hours, mortality can reach 50%.

Although JGI has been described previously, our report shows the correlation between different imaging techniques and highlights the value of tomography in detecting a poorly documented complication in patients, namely intestinal ischemia.

## Conclusions

This is a case of JGI with a typical clinical presentation but with a complication that is rarely documented in imaging studies, such as intestinal ischemia in a patient with a history of gastric surgery. Although this is a rare condition, it is essential to maintain a high index of clinical suspicion, prioritize the use of diagnostic imaging, and intervene surgically in a timely manner to improve patient outcomes and reduce morbidity and mortality.
